# Auditory rhythmical cueing to improve gait and physical activity in community-dwelling stroke survivors (ACTIVATE): study protocol for a pilot randomised controlled trial

**DOI:** 10.1186/s40814-020-00605-1

**Published:** 2020-05-19

**Authors:** Patricia McCue, Silvia Del Din, Heather Hunter, Sue Lord, Christopher I. M. Price, Lisa Shaw, Helen Rodgers, Lynn Rochester, Sarah A. Moore

**Affiliations:** 1 Stroke Research Group, Institute of Neuroscience Newcastle University, 3-4 Claremont Terrace, Newcastle upon Tyne, UK; 2 Institute of Neuroscience Henry Wellcome Building, The Medical School, Framlington Place, Newcastle University, Newcastle upon Tyne, NE2 4HH UK; 3 The Newcastle upon Tyne Hospitals NHS Foundation Trust, Royal Victoria Hospital, Queen Victoria Road, Newcastle upon Tyne, NE1 4LP UK; 4 Stroke Northumbria, Northumbria Healthcare NHS Foundation Trust, Rake Lane, North Shields, Tyne and Wear, NE29 8NH UK; 5 Auckland University of Technology, 55 Wellesley St E, Auckland, 1010 New Zealand

**Keywords:** Stroke, Gait and exercise, Auditory rhythmical cueing, Feasibility, Randomised controlled trial

## Abstract

**Background:**

Mobility problems are present in 70–80% of stroke survivors and can result in impaired gait and reduced physical activity limiting independent living. Auditory rhythmic cueing (ARC) has been used to provide auditory feedback and shows promise in improving a variety of walking parameters following stroke. The aim of this pilot study is to assess the feasibility of conducting a multi-centre, observer blind, randomised controlled trial of auditory rhythmical cueing (ARC) intervention in home and community settings in North East England.

**Methods:**

This pilot observer blind randomised controlled feasibility trial aims to recruit 60 participants over 15 months from community stroke services in the North East of England. Participants will be within 24 months of stroke onset causing new problems with mobility. Each participant will be randomised to the study intervention or control group. Intervention treatment participants will undertake 18 auditory rhythmical cueing (ARC) treatment sessions over 6 weeks (3 × 30 min per week, 6 supervised (physiotherapist/research associate)/12 self-managed) in a home/community setting. A metronome will be used to provide ARC during a series of balance and gait exercises, which will be gradually progressed. The control treatment participants will undertake the same duration balance and gait exercise training programme as the intervention group but without the ARC. Feasibility will be determined in terms of recruitment, retention, adverse events, adherence, collection of descriptive clinical and accelerometer motor performance data at baseline, 6 weeks and 10 weeks and description of participant, provider and clinical therapists’ experiences. As well as using questionnaires to collate participant views, qualitative interviews will be undertaken to further understand how the intervention is delivered in practice in a community setting and to identify aspects perceived important by participants.

**Discussion:**

The ACTIVATE study will address an important gap in the evidence base by reporting whether it is feasible to deliver auditory rhythmical cueing in the home and community to improve gait and balance parameters following stroke. The feasibility of the study protocol will be established and results will inform the design of a future multi-centre randomised controlled trial.

**Trial registration:**

Trial register: ISRCTN, Trial identifier: ISRCTN10874601: Date of registration: 12/03/2018.

## Background

Mobility problems are present in 70–80% of stroke survivors [1], often resulting in impaired gait, which persists despite rehabilitation [2], and reduced physical activity [3]. There are 1.2 million stroke survivors in the UK alone [4], equating to around 840,000 to 960,000 people currently struggling with mobility problems. Gait impairments commonly observed include reduced walking speed, decreased stride length/cadence [5, 6] and increased temporal asymmetry [7], which limits home and community ambulation and is associated with increased dependency and reduced quality of life [8]. Discovering treatments that target gait impairment, balance and mobility are viewed as one of the top priorities in life after stroke [9].

A recent systematic review [10] examined the use of auditory rhythmical cueing (ARC) in motor rehabilitation. ARC provides auditory feedback usually delivered via music or metronome [11, 12] to improve gait and physical activity post-stroke. This review found that stroke patients showed significantly greater walking velocity and improvements in cadence and stride length after intervention with ARC compared to control groups receiving traditional rehabilitative or other types of intervention. Several other systematic reviews on the use of ARC in motor recovery after stroke reported promising results finding improvements in selected gait parameters [2, 11, 13]. The benefit of ARC-based gait interventions include implementation during functional tasks in home and community settings, sustained walking practice in urban and rural environments and overall increased task practice (a recognised key component in recovery post-stroke) [12, 14].

Much of the work to date on ARC has been ward or laboratory based, with many studies using treadmill walking [2, 15], limiting application of findings to ‘real world’ ambulation. One recent study however has addressed the feasibility and potential efficacy of a home-based ARC training programme for stroke survivors and reported that this was feasible and well tolerated [16]. However, whilst finding improvements in walking and functional mobility, the training involved stepping in place/marching on the spot and these activities may not translate to community mobility. Although targeting gait in the home is important, walking in the community is a key activity for stroke survivors which, for many, is still unachievable and as a result they are confined to home [17, 18]. Developing an ARC programme that is deliverable in the home and community may assist more individuals with stroke to mobilise safely beyond their front door.

Resources for therapy-led rehabilitation are often time limited and vary across the UK [19]. Alternative approaches need to be sought for long-term therapy management post-stroke. Technology such as ARC shows promise in bridging the gap [2, 11]. The majority of previous ARC interventions have been delivered face-to-face leading to high costs. Incorporating self-management into an ARC training programme and encouraging self-monitoring, adherence and self-regulation could provide a more economical method of delivering this intervention.

In this pilot study, we aim to determine it is possible to deliver the study protocol over multiple sites in the home and community. This pilot study will be able to inform a future multi-centre observer blind parallel group randomised controlled trial (RCT) of the ARC treatment.

### Research aim

The primary aim of the ACTIVATE study is to determine the feasibility of a multi-centre (*n* = 4), observer blind, parallel group randomised controlled trial of ARC training to improve gait and physical activity after stroke.

### Study objectives

Primary objectives:

1. To identify monthly recruitment rates to determine whether it is possible to enrol at least one patient per month from each study centre.
2. To report participant adherence to ARC intervention and control treatment arms (provider observation and exercise diaries).
3. To assess the acceptability and completeness of proposed methods of data collection to ensure they are feasible for a definitive trial.

Secondary objectives:

4. To report participant retention to the study.
5. To report the success of outcome assessor blinding to participant treatment allocation.
6. To report adverse events in control and intervention treatments during the study.
7. To report the views of study participants about undertaking the ARC intervention at home and in the community.
8. To report the views of providers and clinical therapists about the study protocol.
9. To gather data to allow reporting of summary statistics to allow estimation of the sample size requirements for the definitive trial.

### Progression criteria

Progression criteria have been set based upon recruitment, treatment adherence and data completeness to determine whether progression to a definitive trial is appropriate. These criteria and the traffic light system applied are based upon recommendations from Avery et al. [20].

Potential success criteria:

1. *Recruitment of participants*: green = an average of at least four patients per month recruited across the four sites; amber = an average of at least three patients per month recruited across the four sites; red = two or fewer patients recruited per month across the four sites.
2. *Treatment adherence*: green = an average of at least 80% (840 out of 1080) supervised and self-monitored treatment sessions completed across the intervention and control treatment cohorts measured by provider observation and exercise diaries; amber = an average of at least 70% (756 out of 1080) supervised and self-monitored treatment sessions completed by participants; red = an average of 70% or less supervised and self-monitored treatment sessions completed by participants.
3. *Data completeness:* green = completion (no missing data) of over 85% of key outcome measures (7 day accelerometer measurement; walking speed in the home; miniBEST and self-completion questionnaires: for full description see the ’Baseline assessment’ section) at 10-week outcome assessment for all those completing the treatment programmes; amber = completion of over 70% of key outcome measures at 10 weeks; red = completion of 70% or less of key outcome measures at 10 weeks.

Whilst these criteria will be used to evaluate the likely feasibility of a larger trial, they will primarily be employed to optimise the study design and identify/address issues in order to take remedial steps in a timely fashion. An example of this would be problems around recruitment.

## Methods

### Study design

This study is a pilot multi-centre, observer blind, parallel group randomised controlled trial (RCT), with an allocation ratio of 1:1.

### Study setting

Participants will be recruited from four NHS community stroke services in the North East of England and from regional community stroke group meetings. The study treatments will be delivered in the participant’s homes and nearby community.

### Study population

Eligibility criteria:

*Inclusion criteria*

- Adults (≥ 18 years) with any stroke subtype.
- Within 24 months of stroke onset.
- Able to mobilise independently > 10 m with/without stick indoors but presenting with a gait-related mobility impairment (e.g. gait asymmetry, reduced walking speed, reduced balance, reduced walking confidence based on clinical observation or patient subjective feedback) that would potentially benefit from this intervention.
- Community dwelling and living within the community services catchment area of a participating study centre.
- Able to provide informed consent to participate in the study.

*Exclusion criteria*

- Currently undertaking any active physiotherapy.
- Unable to follow study treatment due to significant cognitive impairment or communication difficulties.
- Diagnosis likely to interfere with adherence to treatment or predispose to falls, e.g. uncorrected hearing problems; registered blind; severe visual/inattention problems as a result of stroke; upper limb impairment restricting use of cueing device; able to mobilise 10 metres but extremely slow gait speed limiting intervention adherence.
- Other neurological or orthopaedic conditions affecting gait (e.g. Parkinson’s disease, multiple sclerosis, osteoarthritis, rheumatoid arthritis and back pain), cardiopulmonary conditions which alter walking ability (e.g. chronic obstructive disorders, angina pectoris) and palliative treatment.

### Sample size

We aim to recruit 60 participants in 15 months at a rate of one patient per trial centre per month. This sample size has been selected as it is the sample recommended for pilot studies [21]. We believe that this is a realistic recruitment rate informed by previous trials [22, 23].

### Case ascertainment recruitment and consent

Eligible patients will be identified by healthcare professionals or by National Institute for Health Research (NIHR) Clinical Research Network (CRN) Clinical Trial Officers (CTO) working within NHS stroke services. CTO’s are part of the hospital stroke team, and there is regular liaison between themselves and other stroke staff to identify patients who may be eligible to be invited to participate in stroke research studies.

Eligible patients will be approached by a healthcare professional or a CTO, a discussion about the study held and an information sheet provided. After allowing sufficient time for this information to be considered, and an opportunity to ask questions, if the eligible patient wishes to take part, they will be asked if they are happy for their details to be shared with the study team. The study team will be alerted and they will contact the patient to provide more information about the study if necessary and arrange a visit to obtain consent in writing. Where a patient is able to provide consent but is unable to sign the consent form (e.g. because of weakness of the dominant hand following stroke), consent will be confirmed orally in the presence of a witness (an individual not otherwise involved in the trial) who will sign the consent form on behalf of the participant.

We will also seek to recruit via advert from community stroke groups within the trial catchment area. On expression of interest, and if eligible, those responding to the advert will be given a participant information sheet and an ‘invitation’ card with study contact names and phone numbers. Their contact details will be taken, and after being given time to consider participating, the study team will contact the potential participant to provide more information about the study and arrange a visit to obtain consent in writing.

**Fig 1:**
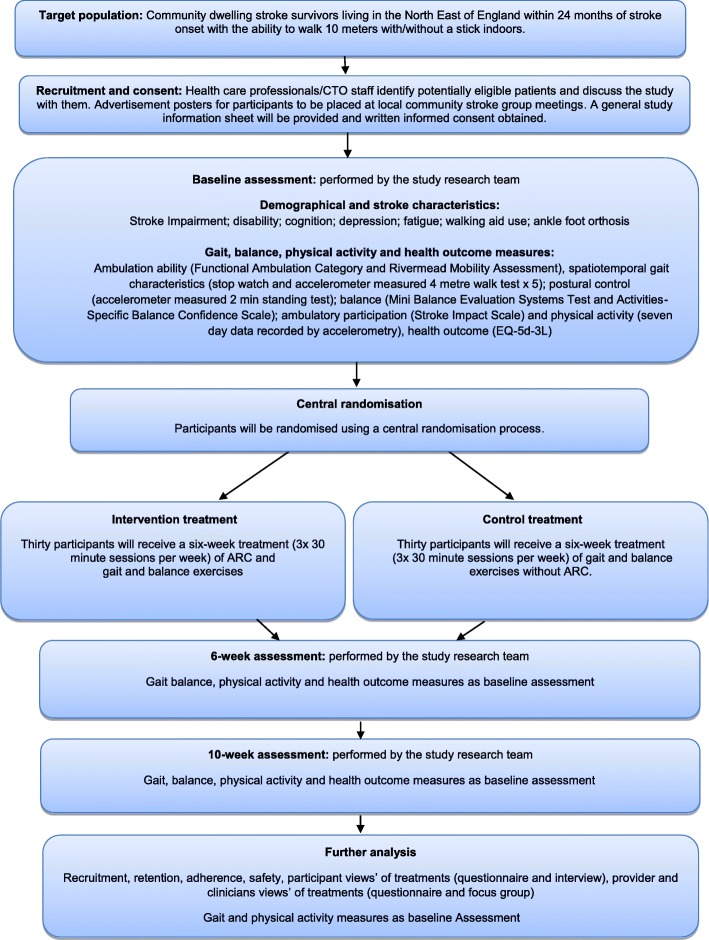
Study Summary

The original consent form will be retained at the study team base. A copy of the consent form will be filed in the medical notes at each study site and a further copy given to the participant. The schedule of events is shown in Table 1 lists the assessments at each study time point, with a further detailed study summary in Fig. 1.

**Table 1:**
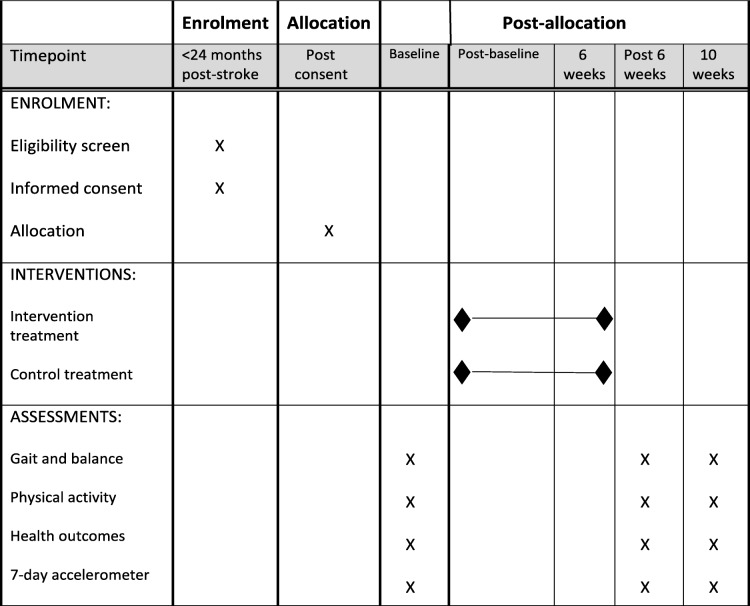
Activate schedule of enrolment, interventions and assessments

Due to the nature of this study and its small size, we plan for the information sheet and consent form to be available only in English. However, interpreters and translation of written material will be considered should a potentially eligible patient require this.

### Loss of capacity to consent to research during participation in the study

The participants in this research study will be stroke patients. It is possible that they may temporarily (e.g. because of intercurrent illness) or permanently (e.g. because of further stroke) lose the capacity to consent to this research project. In either case, it is unlikely that they will be able to continue to participate in a study which involves self-directed practice. In the event of likely temporary incapacity, the ‘intervention’ or ‘control’ treatment will be stopped whilst the participant is unwell but restarted on recovery if the participant wishes to continue. In the event of permanent incapacity, the participant will be withdrawn from the study. Data collected prior to withdrawal will be retained and used in the study analysis as documented in the patient information sheet.

### Study treatments

#### Development of intervention

To develop the ARC intervention we undertook user workshops and an acceptability before and after study. We conducted two user workshops with healthcare professionals and stroke survivors exploring the choice of study metronome, study intervention and control treatment programme content, design of participant handouts/videos and study inclusion/exclusion criteria. Some issues were highlighted, for example clarity with regard to the inclusion/exclusion criteria, and changes implemented accordingly prior to commencement of the before and after study.

Twelve stroke survivors took part in the before and after study (eight receiving the intervention treatment and four receiving the control treatment programme). The study tested the study intervention and control treatment programmes proposed for the main pilot RCT and research protocols including outcome assessment. The treatment and research protocols were feasible and acceptable to stroke participants and providers. Adherence to the intervention was good, and no intervention-related adverse events were reported. The results of the user workshops and the before and after study have informed the design of the ACTIVATE pilot RCT.

### Study intervention treatment: auditory rhythmical cueing and gait and balance exercises

#### Dose and duration

Participants will be asked to undertake three × 30-min ARC treatment sessions per week for 6 weeks (total 18 sessions). This dose and duration has been selected based on findings from previous laboratory-based studies investigating cueing after stroke [11] and on findings from our previous successful RCT in Parkinson’s disease [24].

#### Materials

An auditory rythmical cueing device will be used. The auditory rythmical cue will be delivered through the metronome/phone speaker or through an earpiece attached to the metronome or phone. Depending on the participants’ choice, they will be provided with either a standard metronome (Metro Tuner MT-100 by Musedo with a tempo range of 30–250 bpm) or they will be shown how to download a free metronome app onto their phone (‘ZyMi Metronome’ for the Android operating system or ‘Pro Metronome’ for IOS).

#### Manuals

Participants will be provided with both paper- and web-based password-protected video-based training manuals on how to undertake the ARC treatment. Participants will also be given an exercise and falls diary.

#### Procedures

During the 6-week treatment programme, participants will be taught how to safely undertake a variety of balance and gait exercises using the ARC device (see Table 2 for examples of balance and gait exercises). The ARC treatment will gradually be progressed over the course of the 6 weeks. In the first session, the treatment provider will show the participant how to use the ARC device and at what frequency the ARC device should be set. The frequency of the auditory cue will be dependent on the type of exercise and the needs of the participant. During walking, the metronome frequency will initially be set at the participant’s self-selected stepping frequency as this has been shown to be the most effective method of cueing in stroke [25]. The auditory cue will have a regular pattern. Each step will be cued, rather than only the affected or unaffected lower limb, as this approach has been shown to lead to stronger auditory motor synchronisation [15]. A single tone rather than a separate tone to cue both legs will be used as this approach has been found to be most preferable in stroke [26].

**Table 2:** Examples of exercises included in the ARC training programme

Exercise	Repetitions/time	Progressions
**Balance exercises**		
1. **Weight shift side to side with ARC device**	3 × 10	• Without holding on • Increase the cueing frequency by 5% to work on speed • Reduce the cueing frequency by 5% to work on control • Work on movement selectivity
2. **Weight shift forward and back with ARC device**	10 with the left foot in front 10 with the right foot in front	• Without holding on • Increase the cueing frequency by 5% to work on speed • Reduce the cueing frequency by 5% to work on control
3. **Stepping forwards and backwards with ARC device**	10× forward and back with both feet leading with the right leg 10× forward and back with both feet leading with the left leg	• Without holding on • Increase the cueing frequency by 5% • Reduce the cueing frequency by 5% to work on control
4. **Side stepping with ARC device**	Continue side stepping for 2 min	• Without holding on • Increase the cueing frequency by 5% • Reduce the cueing frequency by 5% to work on control
5. **Turning 180° in both directions with ARC device**	5× in one direction 5× in the other direction	• Increase the cueing frequency by 5% • Reduce the cueing frequency by 5% to work on control • Increase the number of repetitions • Work on the quality of the movement pattern Balance training Walking
6. **Turning 360° with ARC device**	3× in one direction 3× in other direction	• Increase the cueing frequency by 5% • Reduce the cueing frequency by 5% to work on control • Increase the number of repetitions
7. **Forward stepping onto a step with ARC device**	10× forward and back with both feet leading with the right leg 10× forward and back with both feet leading with the left leg	• Increase the cueing frequency by 5% • Reduce the cueing frequency by 5% to work on control • Increase the number of repetitions
8. **Side stepping onto a step with ARC device**	10× stepping both feet onto the step and onto the other side and returning to start position	• Increase the cueing frequency by 5% • Reduce the cueing frequency by 5% to work on control • Increase the number of repetitions
**Gait exercises**		
1. **Standing march with ARC device**	1 min	• Increase time • Increase the cueing frequency by 5% • Reduce the cueing frequency by 5% to work on control
2. **Walking and turning with ARC device**	10 short walks	• Increase time • Increase the cueing frequency by 5% • Reduce the cueing frequency by 5% to work on control • Walking and turning • Walking backwards • Walking whilst carrying an object, e.g. cup
3. **Manoeuvring between objects with ARC device**	Repeat each circuit 5 times (e.g. manoeuvring between two chairs and returning to start position)	• Increase number of circuits • Increase the cueing frequency by 5% • Reduce the cueing frequency by 5% to work on control • Change the objects to make them more difficult to manoeuvre around
4. **Basic community walking with ARC device**	5 min	• Increase time/distance walked • Increase the cueing frequency • Up and down curbs
5. **Advanced community walking with ARC device**	5 min	• Walking in busy areas, e.g. shopping mall • Walking on different surfaces, e.g. grass/sand • Up and down hills

One session of the ARC treatment per week will be supervised by the treatment provider, and during this session, ARC exercises will be progressed and new exercises added dependent on the needs of the participant. During the final 3 weeks of the treatment programme, supervised sessions will focus on ARC training in community settings. A menu of verbal cues will be given to the provider supervising the programme to ensure consistency of cues. To increase motivation, once participants have mastered stepping in time to the beat and are confident with the exercises, they will have the option of using music set at the same cueing frequency during their training sessions. Participants who opt to exercise to a music beat rather than the metronome will be provided with a track list of pieces of music with the correct beat and will be allowed to self-select their own music.

#### Mode of delivery

The ARC intervention treatment will be delivered by two providers (a research physiotherapist and a research associate). The treatment providers will supervise six of the ARC treatment sessions over the 6 weeks. The other 12 ARC treatment sessions will be undertaken independently by participants with telephone support from the treatment providers as necessary (maximum 6 telephone calls). All exercises will initially be supervised in order to determine whether participants are safe to undertake the exercises independently. All community walking sessions with the ARC device will be supervised to ensure safety.

### Study control treatment: gait and balance exercises

#### Dose and duration

Control participants will undertake a matched dose of balance and gait exercise treatment sessions to the intervention treatment group, e.g. three × 30-min sessions per week for 6 weeks (total 18 sessions).

#### Materials

Participants will be provided with paper- and web-based password-protected video-based training manuals on the control treatment programme. Participants will also be given an exercise diary to report adherence and a falls diary.

#### Procedures

Control participants will undertake the same exercises and progressions as the ARC intervention treatment group but without the ARC device and progressions specific to the ARC treatment programme, e.g. increasing the cueing frequency. Participants will be given basic instructions on the exercises but no cues of an auditory nature, e.g. any verbal timing cues whilst they undertake the exercises.

#### Mode of delivery

The control treatment will be delivered by two providers (a research physiotherapist and a research associate). In order to attention match, the control group will be contacted either face to face or by telephone once a week depending on the needs of the participant to check on the treatment progression. The other 12 control treatment group sessions will be undertaken independently with telephone support as necessary (maximum six phone calls over the 6-week period).

### Baseline assessment

A baseline assessment will be performed by a member of the study team following consent to study participation.

To characterise the cohort and inform treatment development, the following data will be collected:

*Demographic information*: Age, sex, pre-morbid function (Modified Rankin Scale) [27] pre-morbid walking status (with/without stick).

*Stroke information*: Date of stroke; stroke type (Ischaemic/haemorrhage); stroke classification subtype (TACS, PACS, LACS, POCS) [28].

The following measures will be taken at study baseline:

*Stroke-related characteristics*: Stroke impairment (National Institute of Health Stroke Scale) [29]; disability (Modified Rankin Scale) [27]; cognition (Montreal Cognitive Assessment) [30]; depression (Physical Health Questionnaire-9) [31]; fatigue (Fatigue Assessment Scale) [32]; walking aid use (with/without stick), ankle foot orthosis (yes/no, type).

*Gait, balance and health outcome measures*: Ambulatory ability (Functional Ambulation Category [33] and Rivermead Mobility Assessment) [34] spatiotemporal gait characteristics including gait speed, asymmetry and variability (4 metre walk test (× 5) recorded both manually and objectively with an AX3 accelerometer (see full description of AX3 below); postural control (2-min standing test objectively measured with an AX3 accelerometer) [35]; balance and gait (Mini Balance Evaluation Systems Test [36] and Activities-Specific Balance Confidence Scale) [37]; participation (Stroke Impact Scale) [38]; health outcome (EQ-5D-3 L) [39].

#### Seven-day measurement of gait and physical activity

At the baseline assessment, participants will be provided with an AX3 accelerometer to wear for 7 days before starting either the control or intervention treatment. The accelerometer will be collected by the treatment provider at the first session. Data on spatiotemporal gait characteristics and broader volume, pattern and variability of walking activity will be quantified using methods previously described [35].

#### Accelerometer description

The AX3 is a single tri-axial accelerometer-based wearable (AX3, Axivity, York, UK, https://axivity.com/, dimensions 23.0 mm × 3.25 mm × 7.6 mm). The AX3 weighs 11 g, has a memory of 512 Mb and a battery life of 14 days. AX3 data capture is 100 Hz (16-bit resolution) at a range of ± 8 g. Recorded AX3 accelerations store locally on the device’s internal memory and then downloaded upon the completion of each walking trial. The feasibility, validity and reliability of the AX3 accelerometer has been previously demonstrated for measuring spatio-temporal aspects of gait and physical activity in a stroke population in the clinic and the community [35].

### Randomisation

Sealed envelope centralised computer-based randomisation service (https://www.sealedenvelope.com/) will be used for randomisation. Allocation concealment will be ensured, as the randomisation code will not be allocated until after the participant has been consented into the trial. Randomisation will take place after all baseline outcome measures have been completed. Once baseline assessments have been completed by the assessors, the providers of the study treatments will use the sealed envelope randomisation service to randomise participants 1:1 into either the ARC intervention or control treatments. Simple randomisations will be used. The randomisation service will be used to number participants.

### Outcome assessments—6-week

The following outcomes will be gathered at the end of the 6-week treatment period.

#### Gait, balance, physical activity and health outcome measures

Gait and balance measures collected at baseline will be repeated (see the “Baseline assessment” section).

#### Seven-day measurement of gait and physical activity

On completion of the treatments, participants will be provided with an accelerometer to wear for 7 days. Participants will be asked to send the accelerometer back to the research team after the 7 days in a pre-paid envelope.

### Outcome assessments—10-week

#### Gait, balance and health outcome measures

Gait, balance and health outcome measures collected at baseline and 6 weeks will be repeated, in order to assess retention of post-treatment effects.

### Seven-day measurement of gait and physical activity

On completion of this assessment, the participants will be provided with an accelerometer to wear for 7 days. Participants will be asked to send the accelerometer back to the research team after the 7 days in a pre-paid envelope.

### Participant and provider views of the intervention

#### Participant views of the treatments

A questionnaire with a combination of closed and open questions will be provided to each of the participants developed with guidance taken from a previous feasibility study of physical activity after stroke via self-management [40].The questionnaires will either be collected by the study providers or returned in pre-paid envelopes.

#### Providers’ views of the treatments

The therapist/researcher who has provided both the treatments will complete a questionnaire with a combination of closed and open questions specifically designed for this study in order to ascertain the feasibility of the treatments.

### Qualitative interviews

In order to expand on the findings of the pilot RCT, a qualitative study will also be undertaken. The views of at least five ARC intervention participants will be sought, via interview, alongside the questionnaire data. These interviews will be separate to the main study and undertaken after the 10-week outcome assessments have been completed. Opinions on various aspects of the study will be sought from the first five intervention participants, or until data saturation has been reached.

#### Qualitative analysis

Face-to-face semi-structured interviews of approximately 1 h will be undertaken. From the 30 participants who will have taken part in the ARC treatment programme, an estimated sample of five stroke survivors will be interviewed unless data saturation is reached before this number. Theoretical saturation is defined as being reached when no new themes emerge from the data. If data saturation has not been achieved by this number, further interviews will be conducted. Purposive sampling will be used to recruit participants who were in the ARC intervention group and had completed the study. Purposive sampling has been chosen to ensure a maximum variation sample based on gender, age and stroke-related physical disability.

Potential participants will be invited to take part either by telephone or by email and given a brief description of what the interview might address, such as how they felt about taking part in the study, engagement with the independent training sessions and any impact they perceive on everyday activities. All participants will be asked to provide written informed consent, which will be collected prior to commencing the interview.

Thematic analysis has been chosen to analyse the data as it is a flexible method that allows themes to emerge from the data [41] and has previously been used to analyse data following exercise interventions [42] and physical rehabilitation following stroke [43]. This ‘rigorous thematic approach’ can produce an insightful analysis that answers particular research questions [41]. Thematic analysis is one of the most commonly used methods of analysing qualitative data, as it is simple, less time-consuming and has a flexible approach.

### Clinical therapists’ views of the intervention

A workshop will be held with clinical therapists from a range of local stroke services to provide feedback on the ARC and control treatments delivered during the RCT. One therapist from each of the study sites will also be invited to shadow the delivery of the ARC intervention during the study to allow for feedback to inform a future multi-centre trial.

### Study withdrawal

No specific study withdrawal criteria have been set. Participants may withdraw from the study at any time for any reason. Should a patient decide to withdraw from the study, a reason for withdrawal will be sought but patients can chose to withdraw without providing an explanation. If a participant decides to withdraw, it will not affect the normal care they receive. Data collected prior to withdrawal will be used in the study analysis unless consent for this is specifically withdrawn.

If a participant does not wish to continue to complete the treatments, they will be asked if they are willing to continue to attend outcome assessments.

Clinical teams, local treatment providers or investigators may also withdraw participants from the study at any time if they feel it is no longer in the participant’s interest to continue, for example, because of intercurrent illness.

### Safety

The safety of the intervention will be evaluated by examining the occurrence of adverse events and falls.

### Falls

Falls diaries will be given to each participant to complete. The providers (a research physiotherapist and a research associate) will check these weekly during face-to-face visits, and any falls between the 6 and 10-week outcome measures will be confirmed by the outcome assessor. Throughout the trial, the following definition of a fall will be applied: ‘A fall is described as an event in which the participant has lost their balance and landed on the floor or ground or lower level, including a slip or trip’ [44]. The falls diaries will be collected by the study team on the final outcome assessment visit.

### Safety evaluation

The standard definitions for adverse events will be used in this study:

#### Adverse event

Any untoward medical occurrence in a subject to whom a study intervention or procedure has been administered, including occurrences which are not necessarily caused by or related to that intervention. An adverse event (AE), therefore, does not necessarily have a causal relationship with the treatment. In this context, ‘treatment’ includes all interventions (including comparative agents) administered during the course of the study. Medical conditions/diseases present before starting study treatment are only considered adverse events if they worsen after starting study treatment.

#### Related AE

Related AE is an AE that results from administration of any of the research study procedures. All AEs judged by either the reporting investigator or the sponsor as having reasonable causal relationship to a study procedure qualify as ‘related adverse events’. The expression “reasonable causal relationship” means to convey in general that there is evidence or argument to suggest a causal relationship.

#### Causality

The assignment of the causality should be made by the investigator responsible for the care of the participant. All adverse events judged as having a reasonable suspected causal relationship to a study procedure are considered to be related adverse events. If any doubt about the causality exists, the local investigator (PI) should inform the chief investigator. In the case of discrepant views on causality between the investigator and others, all parties will discuss the case. In the event that no agreement is made, the main REC and other bodies will be informed of both points of view.

#### Serious adverse event

Serious adverse event (SAE) is an untoward occurrence that:

- Results in death
- Is life-threatening (refers to an event in which the subject was at risk of death at the time of the event; it does not refer to an event which hypothetically might have caused death if it were more severe)
- Requires hospitalisation, or prolongation of existing hospitalisation
- Results in persistent or significant disability or incapacity
- Consists of a congenital anomaly or birth defect
- Is otherwise considered medically significant by the investigator

Medical judgement should be exercised in deciding whether an AE is serious in other situations. Important medical events that are not immediately life-threatening or do not result in death or hospitalisation but may jeopardise the patient or may require intervention to prevent one of the other outcomes listed in the definition above, should also be considered serious.

#### Unexpected adverse event

Unexpected adverse event is an adverse event that is not an expected occurrence in the circumstances of this trial.

#### Recording and reporting of adverse events

This study will only report adverse events which are considered to be serious.

Serious adverse events *exclude*:

- Pre-planned hospitalisations
- Scheduled treatment for pre-existing conditions.

The capture of potential SAEs will take place at the study outcome assessments by including the following questions in the outcome proforma: “are there any new medical problems since the last study assessment?” In addition, we will specifically enquire about falls in response to the participants falls diaries. For any events which fulfil the criteria to be a SAE and are unreported, the study SAE form will be completed.

Events considered to be SAEs will subsequently be documented onto a separate study SAE form, and a causality and expectedness assessment will be performed. As study investigators or other members of the research team may become aware of SAEs at times other than at outcome assessment appointments, the SAE form will also be used to directly capture these events.

Initial/provisional SAE reports can be made by telephone or email to the study co-ordinating centre. All initial/provisional reports must be followed by a fully completed SAE form. If incomplete information is available at the time of this initial report, further information must be provided on a follow-up form as soon as it is available. All SAEs regardless of causality or expectedness will be reported to the chief investigator and trial sponsor (Northumbria NHS Foundation Trust) in line with local policies. The main REC will be notified of related and unexpected SAEs within 15 days of the chief investigator becoming aware of the event.

### Fidelity

The providers of the intervention and control treatments will be trained in the delivery via a face-to-face training session and through provision of a handbook to standardise treatment delivery. Alongside the handbook, providers will have a catalogue of videos of the delivery of the treatments to aid delivery skills.

Providers will be observed by an external member of the study team to ensure adherence to treatment delivery, accommodate for provider differences and prevent provider drift. These observations will be made once every month during the first 3 months of the trial. They will then be observed once every 3 months for the rest of the trial. Providers will keep written records of treatment delivery to ensure delivery of the correct dose of treatment.

In order to ensure participant receipt and correct enactment of the treatments, participants will be shown each exercise, and then asked to describe and demonstrate the exercise back to the provider. Participants will also be asked to confirm how many ARC exercises they have been asked to undertake and will be provided with an exercise diary to document enactment of the exercises. The ARC exercise diary will be collected by the study team on the final outcome assessment visit. The outcome assessor will also be observed whilst conducting outcomes measures on three occasions across the duration of the study.

### Study data collection

All study data will be entered locally by the study team onto a secure online database (MACRO) maintained by Newcastle Clinical Trials Unit. Pseudo-anonymised participant identification codes will be used. Study paper CRF’s will be kept securely in the local investigator site file.

### Blinding

This study will be single blinded. Outcome assessments will be performed by researchers blinded to treatment allocation. After each assessment, the researcher will be asked to record whether they have unintentionally become aware of treatment allocation. Success of outcome assessment blinding will be reported. Emergency un-blinding will not be required for this study.

### Data analysis

Objectives 1–3: Recruitment will be described as rate/month (total and per site). Adherence will be described using data from the exercise diaries (number of planned sessions completed). Acceptability and completeness of methods of data collection will be described using percentages from both treatments.

Objective 4: Retention will be described as percentage from both treatments.

Objective 5: The success of outcome measurement blinding will be described (%).

Objective 6: Adverse events pertaining to the study for each treatment will be described (number of events).

Objective 7: The views of study participants about undertaking the ARC treatment at home and in the community will be described. Summary statistics will be used for answers to closed questions and open questions will be thematically analysed.

Objective 8: The views of providers and clinical therapists about delivery of the study treatments will be described. Summary statistics will be used for answers to closed questions and open questions will be thematically analysed.

Objective 9: As this is a pilot study, statistical comparisons of clinical outcomes (e.g. dynamic gait index, see above) and accelerometer data between treatments will not be undertaken and data will be presented as summary descriptive statistics. To determine which outcome measures to take forwards to a main trial, we plan to review data completeness, patient/staff feedback about acceptability and any new emerging literature about most appropriate measures, to inform decisions. This review will also determine the most appropriate primary outcome measure. Data collected for this selected primary outcome measure (e.g. standard deviation) will subsequently be used as appropriate by statisticians to inform a power calculation for a definitive trial.

### Policies, procedures and dissemination

#### Lone worker policy

The study team and intervention providers will follow the relevant Trust and/or Newcastle University lone workers policy when collecting study data and providing therapy in people’s homes.

#### Confidentiality

Personal data will be regarded as strictly confidential. The study will comply with the Data Protection Act, 2018, General Data Protection Regulations and Caldicott Principles. All study records will be kept at research centres and/or Newcastle University with restricted access. All trial documentation will be retained for future audit in line with the sponsor policies. Participants will not be identified in any report or publication arising from this research. Any feedback comments or quotes will be anonymised.

#### Indemnity

NHS Trusts participating in the study have liability for clinical negligence that harms individuals toward whom they have a duty of care. NHS indemnity covers NHS staff and academic staff with honorary contracts conducting the trial for potential liability in respect of negligent harm arising from the conduct of the study. The Northumbria Healthcare NHS Foundation Trust is a sponsor, and through the sponsor, NHS indemnity is provided in respect of potential liability and negligent harm arising from study management. Indemnity in respect of potential liability arising from negligent harm related to study design is provided by NHS schemes for those protocol authors who have their substantive contracts of employment with the NHS and by Newcastle University Insurance schemes for those protocol authors who have their substantive contract of employment with the university. This is a non-commercial study, and there are no arrangements for non-negligent compensation.

#### Data management

A web-based data entry tool will be developed and administered by Newcastle Clinical Trials Unit for the study. A database manager will monitor data quality under supervision of the project team.

#### Data monitoring

A formal data monitoring committee or equivalent body will not be convened, as the study is of minimal risk and considered too small and of short duration to have official monitoring structures. However, there will be continuous ad hoc monitoring undertaken by study management. Safety data will be reviewed at project meetings. The treatment providers will closely monitor the well-being of individual participants. Stopping the study will be considered if the study management advises against continuing, for example study-related serious adverse events or falls. The chief investigator has the ultimate authority to stop or modify the study.

### Data sharing

We will share anonymised data (referenced only with study number) with approved collaborators both nationally and internationally (inside and outside of the EU) for scientifically sound, peer reviewed studies. Data sharing offers a more open approach that allows us to maximise the impact of the study for the health and wellbeing of the population.

Data sharing will be managed by our data management committee according to the following procedures:

1. Collaborators interested in accessing data from the study will send the data management committee an expression of interest, for example, using data request from or via research platforms data portals.
2. The committee will then review the data request. If required, the data management committee may request changes to the proposed study by collaborators. The data management committee may then approve or reject the proposed study.
3. A data use agreement will be drafted and signed by both parties.
4. As agreed by data managing committee and collaborators, and according to signed data agreement forms, anonymised data will be transferred to the collaborators.

Data will be securely transferred to collaborators. Data will be securely stored by collaborators for a fixed duration, as stated in the signed data use agreement. Only anonymous and unidentifiable data will be sent.

### Auditing

Progress and quality of trial delivery via fidelity checks will be monitored prospectively by the project management group at scheduled meetings. As this is a feasibility study, it will not be audited by an independent auditing company.

### Dissemination of results

The data will be the property of the chief investigator and co-investigator(s). Publication will be the responsibility of the chief investigator. The study will be presented at national and international conferences and reported in peer-reviewed journals. Reports will be written for the study sponsor and regulatory bodies. A summary of the results will be sent to study participants. Anonymised data will be provided to research databases as requested (e.g. the Cochrane Collaboration, the Virtual International Stroke Trials Archive (VISTA) to enable future meta-analyses). Yearly reports will be sent to the funder.

## Discussion

The main aim of this RCT is to determine whether delivery of a home/community-based programme of exercises using auditory rhythmical cueing to improve gait and physical activity in stroke survivors is acceptable and feasible. A significant amount of the stroke survivor population reports gait and balance problems severe enough to interfere with their daily functioning and ability to move freely in the community.

The proposed exercise programme is based on gait and balance exercises delivered in the home, with a combination of supervised and self-managed sessions. Although previous studies have assessed ARC interventions, these have been limited in their design by their focus on laboratory settings/treadmill walking or by only assessing patients ‘stepping-in-place’. By expanding and adapting the ACTIVATE before and after study, this study will provide valuable feasibility data on recruitment, delivery of intervention and measuring outcomes over multiple sites and assess whether it is possible to deliver the ARC intervention in the real world, both in the home and the wider community. This will support moving forward with a multi-centre efficacy trial of ACTIVATE.

## Trial status

Patient recruitment and intervention began in November 2018. The ACTIVATE trial has recruited 16 participants out of our target of 60 at the time of submission of this manuscript (April 2019).

Protocol number and date: Version 2 dated 11.12.2018

Protocol Amendment number 1 Authors SAM, PM.

Revision chronology: 11.12.2017 original. 11.12.2018 Amendment number 1

Primary reason for amendment: changes in sections A27-1, A28 and A29 regarding increasing recruitment potential and additional recruitment materials.

## Data Availability

Data sharing is not applicable to this article as no datasets were generated or analysed during the current study.

## Abbreviations

AE: Adverse event
AFO: Ankle-foot orthosis
AR: Adverse reaction
ARC: Auditory rhythmical cueing
CTO: Clinical Trial Officer
CRF: Case report form
CRN: Clinical Research Network
IOS: Internetwork operating system
MoCA: Montreal Cognitive Assessment
NIHSS: National Institute of Health Stroke Scale
NHS: National Health Service
NRES: National Research Ethics Service
PD: Parkinson’s disease
PHQ-9: Patient Health Questionnaire - 9 item
PI: Principal investigator
RCT: Randomised controlled trial
R&D: Research and Development
REC: Research Ethics Committee
SAE: Serious adverse event
TUG: Timed Up and Go
VISTA: Virtual International Stroke Trials Archive
